# Value of the triglyceride glucose index combined with body mass index in identifying non-alcoholic fatty liver disease in patients with type 2 diabetes

**DOI:** 10.1186/s12902-022-00993-w

**Published:** 2022-04-15

**Authors:** Nong Li, Huiwen Tan, Aixia Xie, Cheng Li, Xuan Fu, Weiting Xang, Amina Kirim, Xuefang Huang

**Affiliations:** 1 Department of Endocrinology and Metabolism, People’s Hospital of Karamay, Xinjiang, China; 2 Department of Endocrinology Metabolism, West China Hospital of Sichuan University, Chengdu, China; 3 First Affiliated Hospital of Xinjiang Medical University, Ürümqi, China

**Keywords:** Triglyceride glucose-body mass index, Type 2 diabetes, Non-alcoholic fatty liver disease

## Abstract

**Background:**

The triglyceride glucose index combined with body mass index is a new index that reflects the degree of insulin resistance. In this cross-sectional study, we aimed to explore the predictive value of the triglyceride glucose-body mass index (TyG-BMI) in relation to the occurrence of non-alcoholic fatty liver disease (NAFLD) in the Chinese population with type 2 diabetes (T2D).

**Methods:**

We selected 826 patients with T2D who were hospitalized at the Department of Endocrinology and Metabolism of Karamay People’s Hospital from September 2016 to October 2018 for this research. The height, weight, fasting blood glucose, serum insulin, and lipid profiles of the subjects were collected. The liver ultrasound showed any degree of echogenic enhancement of liver tissue and the liver appeared brighter than the renal cortex on ultrasound were considered to be NAFLD. The logistic regression analysis was performed to estimate associations between the triglyceride glucose index (TyG), TyG-BMI index, insulin resistance index (HOMA-IR) and the ratio of the triglycerides to high-density lipoprotein-cholesterol with a diagnosis of NAFLD. The receiver operating characteristic curve method was used to analyze its predictive value for NAFLD.

**Results:**

Results of the logistic regression analysis showed that the odds ratios of NAFLD were 6.535 (3.70–11.53) and 4.868 (2.576–9.200) for the TyG-BMI before and after correction,respectively(*P* < 0.001). The area under the curve (AUC) for TyG-BMI was 0.727 (0.691–0.764), which was the highest among all the other parameters studied.

**Conclusion:**

Compared with the TyG index, the TG/HDL-C and HOMA-IR, the TyG-BMI was a more effective predictor of NAFLD in T2D.

## Backgroud

Non-alcoholic fatty liver disease (NAFLD) to a clinic entity characterized by liver steatosis after exclusion of significant alcohol consumption and other chronic liver diseases. If the disease progresses, it could evolve into liver fibrosis and even liver cancer. Several studies have shown that NAFLD is related to diseases such as type 2 diabetes, abdominal obesity, dyslipidemia, hypertension, and cardiovascular diseases, and is closely related to insulin resistance (IR) and genetic susceptibility to metabolic stress-induced liver injury [1–5]. In addition, mitochondrial dysfunction also plays a key role in the occurrence and development of NAFLD, among which, sirtuin − 4 is a preminent factor as evident in negative regulator of the mitochondria oxidative metabolism [6].

In recent years, with the global epidemic of obesity and metabolic syndrome, the prevalence of NAFLD has gradually increased worldwide [7, 8]. NAFLD is one of the important global public health issues of the twenty-first century. NAFLD affects 30% of adults and 10% of children in the United States [9, 10]. analyzed data available from 867 adolescents from participants 12–18 years old included in the National Health and Nutrition Examination Survey 2017–2018 in the United States. The results show 24.16% of adolescents had any degree of steatosis,11.6% of the adolescents had moderate to severe degree of steatosis, 4.4% of adolescents had significant fibrosis [11]. In the past decade, NAFLD has grown rapidly and has been presenting a trend of younger onset in China. In 2004, the prevalence of NAFLD among adults in Shanghai was approximately 15.4%.In 2011, the prevalence of NAFLD in adult communities in Beijing was up to 35.1% [12, 13] .NAFLD is a chronic liver condition that is gaining more and more importance in China. In a retrospective study assessing the clinical characteristics and initial disease severity of patients with NAFLD and the incidence and risk factors of NAFLD progression [14], 12.3% of NAFLD-free patients showed progression, 24.7% of patients with NAFLD combined with T2DM progressed. The risk of T2DM and disease progression is about twice the risk of T2DM-free disease progression, and the mortality risk increases as the disease progresses. In this study, this association between T2DM and NAFLD was found in nearly 40% of NAFLD patients, further illustrating the importance of diabetes management to reduce the risk and adverse consequences of liver-related death. The study found that screening strategies based on noninvasive scores are able to exclude advanced liver fibrosis in 50–67% of patients with T2DM [15]. Therefore, it is particularly important to screen for NAFLD patients in the T2DM population as well as monitor disease progression of NAFLD.

T2DM and NAFLD are both related to IR. IR refers to a state in which the body exhibits reduced sensitivity and reactivity to insulin [16]. The current “gold standard” for evaluating IR is the euglycemic hyperinsulinemic clamp test. However, as it is a complicated, time-consuming, and labor-intensive test, its wide application in clinical work remains limited. Recent studies have reported that the triglyceride glucose (TyG) index, which is calculated on the basis of triglyceride and fasting blood glucose levels, and the Homeostasis model assessment can be used to identify the insulin resistance index (HOMA-IR). There is a significant correlation between the glucose metabolism rate M value obtained from the euglycemic hyperinsulinemic clamp [17–19], and it has become a reliable proxy for evaluating IR. To date, a few studies have evaluated the relationship between the TyG index and the incidence of NAFLD in populations without diabetes [20–22], but no study has investigated the relationship between the TyG-BMI and incidence of NAFLD in populations with diabetes. In this study, we tried to clarify the relationship between NAFLD and the TyG with BMI in the T2DM populations and explore its predictive value for the occurrence of NAFLD in the T2DM population in China.

## Materials and methods

### Study participants

A total of 826 in-patients who were treated at the Department of Endocrinology and Metabolism, Karamay People’s Hospital from September 1, 2016, to October 31, 2018 were screened for participation in this cross-sectional observational study referring to the practice guidelines of the American Gastroenterology Association and the American Liver Disease Research Association [23]. Patients with type 2 diabetes (T2D) were included in the study. Of the 826 patients, 552 had NAFLD, 274 did not have NAFLD. The following inclusion criteria were applied for patients: 1. Meet the diagnostic criteria for type 2 diabetes [24]; 2. It meets the diagnostic criteria of non-alcoholic fatty liver disease, that is, no history of alcohol consumption or alcohol consumption less than 30 g/ day (female < 20 g/ day), and ultrasound imaging examination meets the manifestations of diffuse fatty liver disease;Patients were screened according to the following exclusion criteria: previous long-term heavy drinking or combined with viral hepatitis, drug-induced liver disease, total parenteral nutrition, hepatolenticular degeneration, autoimmune liver disease and other specific diseases that can lead to fatty liver; inflammatory bowel disease; hypothyroidism; Cushing’s syndrome; β-lipoproteinemia; and insulin resistance-related conditions such as lipoatrophic diabetes and Mauriac syndrome; and other patients with T2D that could cause fatty liver.

### Medical data collection and physical examination

This research project follows the Helsinki Declaration and China’s clinical research management norms and regulations. The research plan was approved by the Medical Ethics Committee of Karamay People’s Hospital. Inpatients had to voluntarily sign informed consent forms before they could be used as research subjects for research data collection. General demographic information and anthropometric measurement data were collected for use in the research. For the demographic information, age, ethnicity, gender, occupation, education level, previous medical history, and personal lifestyle(e.g. alcohol abuse, smoking). For anthropometric measurements, the subjects were asked to fast, take off their shoes and wear light clothing, and height and weight measurements were obtained. The body mass index (BMI) = weight (kg)/height (m)^2^. Blood pressure was measured according to the recommendations of the American Heart Association, and the blood pressure in the right arm was recorded by a qualified investigator (nurse) with a mercury sphygmomanometer. Blood pressure (BP) was measured three times, and the average of the systolic and diastolic blood pressure values was used for analysis.

### Laboratory measurement and index calculation

Venous blood was collected early in the morning after the patient had fasted for at least 8 h. Determination of biochemical indicators: fasting blood glucose, triglyceride, total cholesterol, and high-density lipoprotein cholesterol (HDL-C) levels were measured using the COBAS 8000 chemical analyzer (Roche, Swiss). Plasma insulin levels were measured using an E601 automatic chemiluminescence system (Roche, Germany). On the day of blood collection, blood biochemical indicators were determined at the Medical Test Center of Karamay People’s Hospital, Xinjiang, China. Quantitative analysis of insulin resistance uses the insulin resistance index (HOMA-IR) obtained by the steady-state model to identify insulin resistance. The HOMA-IR is calculated as follows: HOMA-IR = fasting insulin (μU/dL) × fasting blood glucose (mg/dL)/22.5. TyG index [17]: Ln [TG (mg/dL) × fasting blood glucose (mg/dL)/2]. The TyG-BMI represents the TyG index × BMI [25].

### Ultrasound analysis

A color Doppler ultrasound system (IU22, Philips Healthcare, Andover, MA) with a 1.0–5.0 MHz sensor was used to perform an abdominal ultrasound scan to diagnose fatty liver. Abdominal ultrasonography was performed on the subjects by two professionally trained and experienced ultrasound diagnostic physicians in a blinded manner. The examiner was blinded to the clinical information of the subject, and used the echogenicity of the liver tissue, the difference between the liver and the right kidney, and the visibility of the vascular structure to arrive at a diagnosis [26]. In this study, signs of hepatic steatosis were considered to be NAFLD if the liver ultrasound showed any degree of echogenic enhancement of liver tissue or if the liver appeared brighter than the renal cortex on ultrasound. Subjects with NAFLD were classified according to the presence and severity of this disease.

### Statistical analysis

Excel 2007 was used to generate the database, and errors were corrected after double data entry. All statistical analyses were performed using the SPSS 22.0 statistical software package (IBM, Armonk, New York). Continuous data for skewed distributions were expressed as medians and interquartile ranges (IQR) and compared using Kruskal-Wallis H test or the Mann-Whitney U test. Categorical variables were compared using the chi-square test. The Logistic regression was performed to analyze associations between NAFLD diagnosis and the TyG index, HOMA-IR, TYG-BMI, and TG/HDL-C after adjustment for any confounding factors (age, gender, BMI, SBP, DBP, diabetes duration, fasting and postprandial blood glucose).we converted the TyG index, HOMA-IR, TYG-BMI, and TG/HDL-C into ordered multi-classification variables, and we divided them into four classifications based on the quartile of these variables, that is, Q1 was < 25%,Q2 was 25–50%,Q3 was 50–75%, and Q4 was 75% and above, but HOMA-IR was only divided into dichotomy, Q1 was < 75%, and Q2 was 75% and above;We designated Q1 as the reference group and compared the changes in the risk of non-alcoholic fatty liver disease in Q2,Q3, and Q4 groups relative to the reference group and the logistic regression analysis was applied to calculate the TyG index, TyG-BMI, and TG/HDL-C quartiles 2–4. The odds ratio of NAFLD and the 95% confidence interval (CI) for HOMA-IR were compared with the reference value below the 75th percentile. Then, we determined the receiver operating characteristic curve (ROC) of each parameter and calculated the area under the curve (AUC) and compared the AUC between different groups, and We studied the ability of these parameters to predict the occurrence of NAFLD. The point with the highest sensitivity as well as specificity was considered the cutoff point. The difference was statistically significant with a *P* value of < 0.05 (two-tailed).

## Results

### Clinical characteristics of patients

A total of 826 subjects were included in the study. Of these, 274 had no NAFLD and had an average age of 59 (49–67) years, while 552 patients had NAFLD, and had an average age of 55 (47–64) years. Among the patients with NAFLD, There were 375 men, 177 women, and among the patients without NAFLD, there were 178 men and 96 women. There were no statistically significant differences in the gender ratio, SBP, and HbA1C between the two groups (*P* > 0.05); the age, BMI, DBP, and duration of diabetes, fasting blood glucose (FBG), postprandial blood glucose (PBG), HOMA-IR, triglyceride (TG), total cholesterol (TC), high-density lipoprotein-cholesterol (HDL-C), and TyG were significantly different among the groups (*P* < 0.05). The BMI, DBP, FBG, PBG, HOMA-IR, TG, TC, and TyG were significantly higher in patients with NAFLD than in those without NAFLD, while the age, duration of diabetes, and HDL-C were significantly lower in patients with NAFLD than in those without NAFLD (see Table 1).

**Table 1:** Comparison of baseline characteristics between the group with NAFLD and the group without NAFLD

Indicators	NAFLD(*n* = 552)	NON-NAFLD(*n* = 274)	Z/x^2^	*P* -value
Age (year)	55 (47 ~ 64)	59 (49 ~ 67)	− 3.03	0.002
Sex (male/female)	375/177	178/96	−0.854	0.393
BMI (kg/m^2^)	27.2 (24.6 ~ 29.8)	24.2 (22.3 ~ 26.4)	−9.59	< 0.001
SBP (mmHg)	127 (120 ~ 140)	120 (110 ~ 140)	−1.72	0.086
DBP (mmHg)	77 (70 ~ 80)	75 (70 ~ 80)	−2.57	0.010
Diabetic course (year)	7.0 (1.0 ~ 12.0)	9.0 (3.0 ~ 15.3)	−3.43	0.001
HbA_1_C (%)	9.20 (7.9 ~ 10.5)	9.1 (7.0 ~ 10.9)	−1.31	0.190
FBG (mmol/L)	8.9 (7.0 ~ 11.1)	7.4 (6.0 ~ 10.2)	−4.00	< 0.001
PBG (mmol/L)	14.5 (11.4 ~ 18.1)	13.6 (9.5 ~ 16.5)	−3.52	< 0.001
HOMA-IR	2.45 (0.59 ~ 9.88)	0.86 (0.01 ~ 2.59)	−7.19	< 0.001
TG (mmol/L)	1.86 (1.33 ~ 2.67)	1.35 (0.96 ~ 1.95)	−7.40	< 0.001
TC (mmol/L)	4.41 (3.76 ~ 5.04)	4.19 (3.41 ~ 4.97)	−2.59	0.009
HDL-C (mmol/L)	0.96 (0.82 ~ 1.11)	1.04 (0.88 ~ 1.26)	−4.744	< 0.001
LDL-C (mmol/L)	2.86 (2.31 ~ 3.44)	2.69 (2.02 ~ 3.36)	−2.580	0.010
TYG	6.5 (6.2 ~ 6.7)	6.3 (6.0 ~ 6.5)	−7.13	< 0.001

*NAFLD* Non-alcoholic fatty liver disease, *BMI* Body mass index, *SBP* Systolic blood pressure, *DBP* Diastolic blood pressure, *FBG* Fasting blood glucose, *PBG* Blood glucose 2 h after meal, *HOMA-IR* Homeostatic model assessment for insulin resistance, *TG* Triglycerides, *TC* Total cholesterol, *HDL-C* High-density lipoprotein cholesterol, *LDL-C* Low-density lipoprotein cholesterol, *TYG* a product of triglyceride and fasting glucose

### Relevant indicators and risk assessment of the incidence of NAFLDA

The results of the logistic regression analysis showed that compared with participants in the minimum quartile (Q1), the TyG index, TyG-BMI, and Q2–Q4 of the TG/HDL-C ratio had a higher Odds ratio (OR) of NAFLD (Table 2). The TyG-BMI had the highest Odds ratio of NAFLD. Before and after adjustment, the Odds ratio (OR) of Q4 reached 6.54 (95% CI 3.70–11.53) and 4.868 (95% CI 2.576–9.200, *P* < 0.001). The second highest Odds ratio was for the TyG index, with OR values of 3.455 (2.060–5.795) and 3.405 (1.900–6.102), and the Odds ratios of HOMA-IR before and after adjusting for confounding factors were 2.451 (1.539–3.904) and 2.951 (1.732–5.026), respectively (see Table 2).

**Table 2:** Odds ratios and adjusted odds ratios for NAFLD in quartiles of each parameter

Parameters	Unadjusted OR (95% CI)	***P***-value	Adjusted OR (95% CI)	***P***-value
**TyG index**				
**Q1**	1		1	
**Q2**	1.738 (1.071–2.822)	0.025	1.992 (1.142–3.475)	0.015
**Q3**	3.08 (1.859–5.108)	< 0.001	2.942 (1.677–5.163)	< 0.001
**Q4**	3.455 (2.060–5.795)	< 0.001	3.405 (1.900–6.102)	< 0.001
**TyG-BMI**				
**Q1**	1		1	
**Q2**	2.156 (1.348–3.448)	0.001	1.843 (1.096–3.098)	0.021
**Q3**	4.754 (2.802–8.067)	< 0.001	3.661 (2.049–6.539)	< 0.001
**Q4**	6.535 (3.704–11.529)	< 0.001	4.868 (2.576–9.200)	< 0.001
**HOMA-IR**				
**Q1**	1		1	
**Q2**	2.451 (1.539–3.904)	< 0.001	2.951 (1.732–5.026)	< 0.001
**TG/HDL-C**				
**Q1**	1		1	
**Q2**	1.566 (0.972–2.522)	0.065	1.356 (0.788–2.333)	0.271
**Q3**	2.288 (1.393–3.760)	0.001	1.882 (1.079–3.281)	0.026
**Q4**	4.171 (2.435–7.147)	< 0.001	2.772 (1.544–4.976)	0.001

*NAFLD* Non-alcoholic fatty liver disease, *TyG index* a product of triglyceride and fasting glucose, *TyG-BMI* TyG index × BMI; *HOMA-IR* Homeostatic model assessment for insulin resistance; (μU/dL) × fasting blood glucose (mg/dL)/22.5; Q, quartile of these variables, that is, Q1 was < 25%,Q2 was 25–50%,Q3 was 50–75%, and Q4 was 75% and above, but HOMA-IR was only divided into dichotomy, Q1 was < 75%, and Q2 was 75% and above

### Comparison of the parameters to the predictive power of NAFLD

**Fig. 1:**
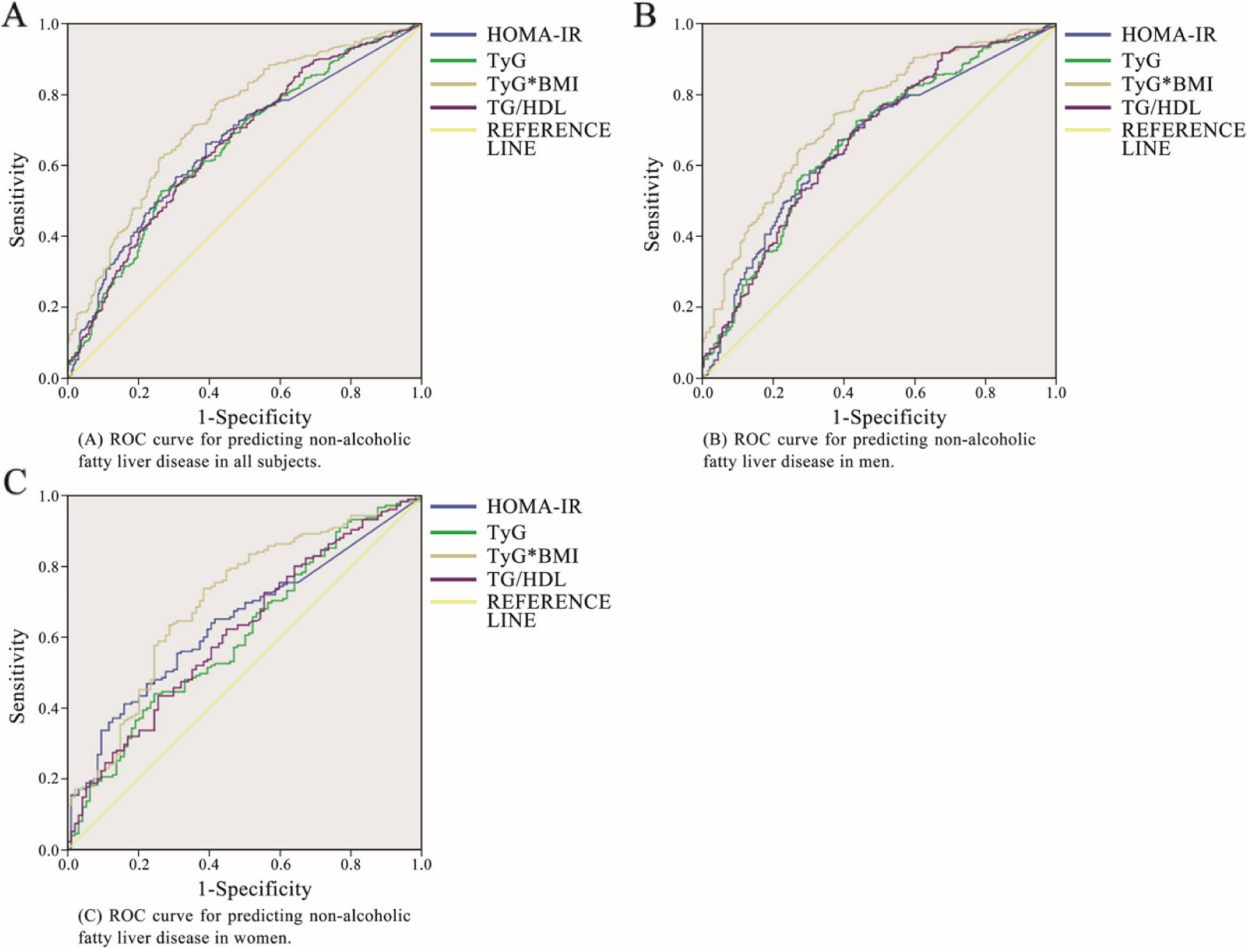
Receiver-operating characteristic (ROC) curve of each parameter for predicting non-alcoholic fatty liver disease

The results of the ROC curve analysis of the TyG index, TyG-BMI, HOMA-IR, and the TG/HDL-C ratio corresponding to 95% CI are shown in Table 3 and Fig. 1. The AUC of NAFLD was the highest for TyG-BMI at 0.727 (95% CI 0.691–0.764), followed by TG/HDL-C (0.657, 95% CI 0.617–0.696), and HOMA-IR (0.655, 95% CI 0.616–0.694), and TyG (0.651, 95% CI 0.611–0.691). In the gender subgroup analysis, the AUCs for the TyG-BMI of men and women with NAFLD were 0.739 (0.695–0.783) and 0.702(0.636–0.768), respectively. In the BMI subgroup analysis,the AUCs for the TyG-BMI of BMI < 25 kg/m^2^ and BMI≧25 kg/m^2^ with NAFLD were 0.671(0.611–0.730) and 0.674 (0.6180–0.730),respectively. When the cutoff value of the ROC curve drawn by the TyG-BMI for NAFLD is 169.92, the sensitivity and specificity of the predicted NAFLD were 62.2 and 73.8%, respectively. The specificity + sensitivity of TyG-BMI, TyG, TG/HDL-C and HOMA-IR (The sum of the specificity and sensitivity of the above parameters)were 136, 126.2, 123.8 and 127%, respectively, Compared with the other three parameters, the sensitivity + specificity value corresponding to the cutoff value of TyG-BMI was the largest. Among men, the most sensitive parameter for predicting NAFLD was TyG, followed by TG/HDL-C and HOMA-IR, and the most specific parameter for predicting NAFLD was TyG-BMI, followed by HOMA-IR and TG/HDL-C. Among women, the most sensitive parameter for predicting NAFLD is TyG-BMI (73.7%), followed by the TyG index and the TG/HDL-C. Furthermore, the most specific parameter for predicting NAFLD was HOMA-IR (84%), followed by the TyG index and the TG/HDL-C ratio (see Table 3 and Fig. 1). The specificity + sensitivity of TyG-BMI, TyG, TG/HDL-C and HOMA-IR (The sum of the specificity and sensitivity of the above parameters)were 136, 126.2, 123.8 and 127%, respectively. The positive and negative Likelihood Ratio for the TyG-BMI of all subjects were 2.374 and 0.512, respectively. Compared with TyG index, HOMA-IR, and the TG/HDL-C ratio,the positive Likelihood Ratio of the TyG-BMI was the highest, the negative Likelihood Ratio was the lowest.

**Table 3:** Areas under the receiver-operating characteristic curves for each parameter for predicting non-alcoholic fatty liver disease

Parameters	Area under the ROC curve	95% CI	*P*-value	SE	Cutoff value	Sensitiyity(%)	Specificity(%)	Positive Likelihood Ratio(+LR)	Negative Likelihood Ratio(−LR)
All subjects									
TyG index	0.651	0.611–0.691	< 0.001	0.020	6.50	52.80	73.40	1.985	0.643
TyG-BMI	0.727	0.691–0.764	< 0.001	0.019	169.916	62.20	73.80	2.374	0.512
HOMA-IR	0.655	0.616–0.694	< 0.001	0.020	1.530	66.10	60.90	1.69	0.557
TG/HDL-C	0.657	0.617–0.696	< 0.001	0.020	1.571	61.80	62.00	1.626	0.616
Male									
TyG index	0.669	0.620–0.718	< 0.001	0.025	6.350	72.40	56.80	1.676	0.486
TyG-BMI	0.739	0.695–0.783	< 0.001	0.022	161.873	74.30	63.10	2.013	0.407
HOMA-IR	0.664	0.615–0.712	< 0.001	0.025	1.530	67.00	61.90	1.759	0.533
TG/HDL-C	0.671	0.622–0.720	< 0.001	0.025	1.567	68.90	58.50	1.660	0.531
Female									
TyG index	0.606	0.535–0.676	< 0.001	0.036	6.50	44.00	75.50	1.796	0.742
TyG-BMI	0.702	0.636–0.768	< 0.001	0.034	154.887	73.70	61.7	1.924	0.426
HOMA-IR	0.642	0.575–0.708	< 0.001	0.034	3.620	41.10	84.00	2.568	0.701
TG/HDL-C	0.615	0.546–0.685	< 0.001	0.036	1.714	43.40	74.50	1.704	0.760
BMI < 25 kg/m^2^									
TyG index	0.654	0.595–0.714	< 0.001	0.030	6.50	53.00	73.72	2.017	0.638
TyG-BMI	0.671	0.611–0.730	< 0.001	0.030	146.074	61.60	72.44	2.235	0.530
HOMA-IR	0.658	0.598–0.718	< 0.001	0.031	1.36	71.34	59.62	1.767	0.481
TG/HDL-C	0.685	0.627–0.744	< 0.001	0.030	1.56	61.59	71.79	2.183	0.535
BMI≧25 kg/m^2^									
TyG index	0.631	0.573–0.689	< 0.001	0.030	6.51	51.97	73.91	1.992	0.650
TyG-BMI	0.674	0.6180–0.730	< 0.001	0.028	170.034	83.73	70.48	2.836	0.231
HOMA-IR	0.629	0.573–0.685	< 0.001	0.028	3.04	50.92	73.04	1.889	0.672
TG/HDL-C	0.590	0.533–0.648	< 0.001	0.029	2.34	41.21	76.52	1.755	0.768

*CI* Confidence interval, *SE* Standard error. **+** LR = Sensitiyity/(1-Specificity); −LR = (1-Sensitiyity)/Specificity

## Discussion

Currently, regarding the pathogenesis of NAFLD, it is believed that the combination of genetic susceptibility to this condition and the presence of multiple factors such as IR, inflammatory factors secreted by adipose tissue, gut microbiota, and specific genetic and epigenetic factors trigger the onset of NAFLD. Of these, IR plays a key role in the pathogenesis of fatty liver, which can cause excessive lipid deposition in liver cells, which is closely related to the occurrence of NAFLD [27–29]. The pathophysiology of NAFLD is IR, which is clinically manifested as metabolic syndrome, i.e., hypertension, hyperlipidemia, central obesity, hyperglycemia, and NAFLD.

The results revealed that the TyG index can effectively identify the risk of IR in Chinese individuals [30]. In the San Antonio metabolism (SAM) study, Gastaldelli et al. [31] proposed that because TyG is closely related to the liver fat mass, it is not a good method to measure peripheral IR, but it is a good method to measure liver IR. In fact, hypertriglyceridemia can increase the transport of free fatty acids to the liver, cause liver fat accumulation, hepatic IR, cause fatty liver, and increase glucose output in the liver. Studies have found that the TyG index calculated on the basis of the TGs and FBG levels can diagnose steatosis, is associated with IR and can predict IR. However, this measurement is confounded by the presence of fibrosis and inflammation, as a result of which steatosis is not accurately quantified [32].

Studies have shown that increasing TG and decreasing HDL-C levels can lead to IR. When the circulating TG levels are high, heparin activates lipoprotein lipase to increase intravascular lipolysis of TG, thereby increasing the risk of tissue exposure to free fatty acids (FFAs). High FFAs can cause IR through oxidative stress pathways [33]. Clinical studies of Caucasian populations have proven that the TG/HDL-C ratio can predict IR, and several studies conducted in China have also shown that TG/HDL-C can predict IR [34, 35].

The relationship between obesity and IR has also been well established, and excess adipose tissue has been shown to promote insulin resistance [36]. Studies have shown that obesity is closely related to liver steatosis. BMI is related to the occurrence of NAFLD in the general population or in specific disease groups such as among patients with hypertension. Furthermore, it has been reported that 65–92.3% of patients with a BMI of > 40 kg/m^2^ have NAFLD, and the higher the BMI in NAFLD patients, the more severe is the case of liver steatosis [34]. The BMI may affect the predicted TyG value for NAFLD.

Therefore, combining the TyG and obesity indices can help better predict the occurrence of IR and NAFLD compared to the TyG index alone. Zhang et al. revealed that after adjusting for potential confounding factors, there is a strong positive correlation between the TyG-BMI and NAFLD risk. The TyG-BMI can accurately identify NAFLD, as the AUC of TyG-BMI was 0.835 (0.824–0.845), which is higher than that of TyG, BMI, TG, FPG, and other components. Thus, TyG-BMI is an effective indicator for identifying the NAFLD patients without obesity. In this study, we compared the efficacy of TyG, TyG-BMI, and four other parameters to predict the occurrence of NAFLD in patients with T2DM. The results revealed that TyG-BMI has an AUC of 0.727 (95% CI, 0.691–0.764) in the accuracy of predicting NAFLD in T2D. The optimal cutoff point for the diagnosis of NAFLD is 169.92. At this time, the sensitivity and specificity of this factor were 62.2 and 73.8%, respectively. To compare the prediction ability of the two models, NetReclassification Index (NRI) is adopted. NRI = (specificity predictor 1+ sensitivity predictor 1) - ((specificity predictor 2+ sensitivity predictor2),If NRI > 0, the predictive ability of predictor 1 was higher than that of predictor 2.The results show the specificity + sensitivity of TyG-BMI was the largest in Table 3.The results suggest that compared with TyG, TG/HDL-C ratio, and HOMA-IR, the combination of TyG index and BMI can better predict the occurrence of T2D and NAFLD in both men and women, and the accuracy of TyG-BMI in predicting NAFLD with type 2 diabetes was also the highest in both men and women.

Likelihood ratio is an indicator reflecting authenticity, which is a compound indicator reflecting both sensitivity and specificity. Likelihood ratio was not affected by prevalence. The positive likelihood ratio is the ratio of the true positive rate to the false positive rate of the screening results. The greater the ratio, the greater the probability of the test result being true positive;Negative likelihood ratio is the ratio of false negative rate to true negative rate of screening results. The smaller the ratio, the greater the probability of true negative when the test results are negative. From the comprehensive data analysis in Table 3,Compared with the likelihood ratio of the other three parameters, the positive likelihood ratio of TyG-BMI is the biggest and the Negative likelihood ratio is the smallest. So,TyG-BMI is the best method for the diagnosis of NAFLD. The results of this study suggest that the predictive value of TYG-BMI for non-alcoholic fatty liver disease in persons with obesity is higher than that in persons without obesity.

Abdominal obesity includes subcutaneous adipose tissue and visceral adipose tissue. Visceral adipose tissue has a greater effect on the IR [36]. Studies have shown that visceral fat produces more FFAs than subcutaneous fat, thereby increasing the risk of IR and diabetes [37]. In addition, visceral fat secretes a variety of inflammatory cytokines and adipokines, which may also promote the occurrence of IR and diabetes [37, 38]. In this study, TyG-BMI was a more accurate predictor of NAFLD in men than in women, which may be related to the fact that male obesity is mostly abdominal obesity. These results also suggest that weight control is more important to prevent non-alcoholic fatty liver disease in men with type 2 diabetes than in women.

Currently, liver biopsies are the best diagnostic and staging methods for nonalcoholic steatohepatitis (NASH) and NAFLD. However, it is invasive, and its associated complications and irregular liver biopsy sampling limit its use. Noninvasive tools for detecting NAFLD include ultrasound, computed tomography, and magnetic resonance spectroscopy. The latter two are expensive and time-consuming tools, and ultrasound is currently recommended as the first-line imaging technique for the clinical screening of NAFLD patients. In addition, researching a simple and effective diagnostic tool that can identify the risk of NAFLD at an early stage will help the early detection and management of such patients, which is very important for public health. The results of this study suggest that the combination of the triglyceride glucose index and body mass index (TyG-BMI) is a good indicator for identifying IR and predicting NAFLD in patients with T2D.

The present study has several limitations. First of all, due to its cross-sectional design, the identified relationship is not forward-looking, and causality cannot be determined. Further prospective cohort studies are needed to determine whether TyG-BMI can predict the future occurrence of NAFLD. Second, due to the lack of waist circumference information, TyG and abdominal obesity indicators could not be combined for analysis and comparison. In addition, the research subjects are from inpatients and the number of cases is relatively small. If there is a large sample of natural populations derived from outpatient examinations and participating in health examinations, the research results may be better. In this study, the diagnosis of nonalcoholic fatty liver disease was determined by ultrasound, which has several limitations; (1) The ultrasonographic manifestations of diffuse hepatic steatosis and diffuse fibrosis are similar and sometimes difficult to distinguish; (2) Liver fat content cannot be accurately quantified (i.e. grade: mild, moderate and severe steatosis); (3) it is an operator-dependant modality with varying results between operators; Therefore, ultrasound diagnosis of non-alcoholic fatty liver disease will also lead to misdiagnosis and missed diagnosis, which will also affect the prediction of TyG-BMI for non-alcoholic fatty liver disease.

This study shows that TyG-BMI is a strong predictor of NAFLD in T2DM patients. This result also suggests that reducing blood TG levels, weight loss, and increased physical activity are important measures that will help prevent NAFLD in T2DM patients. This is also the main management measure to prevent the occurrence of NAFLD in patients with T2D.

## Conclusion

A very high proportion of T2D patients have NAFLD. In our study, nearly 67% of patients with T2D had NAFLD. Therefore, it is worthwhile to screen for effective NAFLD markers in patients with T2D. TyG-BMI is a valuable index for screening NAFLD, and it is an effective noninvasive method to identify NAFLD. To improve the prediction performance of NAFLD in patients with T2D, it can be predicted at a low cost using values obtained from routine laboratory tests. Therefore, we recommend applying the TyG-BMI value to the risk assessment of NAFLD in people with T2D in clinical practice and in future epidemiological studies.

## Data Availability

The dataset used in this study is available and can be provided upon written request (Nong Li,Email:linongklmy@yeah.net).

## Abbreviations

NAFLD: Non-alcoholic fatty liver disease
ROC: Receiver operating characteristics
BMI: Body mass index
AUC: Areas under the curve
CI: Confidence intervals
TyG: Index, a product of triglyceride and fasting glucose
HOMA-IR: The homeostasis model assessment for insulin resistance
